# 1,3-Bis[2-hy­droxy-2-(6-meth­oxy-2,2-dimethyl-3a,5,6,6a-tetra­hydro-2*H*-furo[2,3-*d*][1,3]dioxol-5-yl)eth­yl]-2,3-dihydro-1*H*-1,3-benzodiazol-2-one

**DOI:** 10.1107/S160053681105625X

**Published:** 2012-01-11

**Authors:** Brahim Lakhrissi, Mohamed Massoui, Youssef Ramli, El Mokhtar Essassi, Seik Weng Ng

**Affiliations:** a Laboratoire de Chimie des Agroressources, Université Ibn Tofail, Kénitra, Morocco; b Laboratoire de Chimie Organique Hétérocyclique, Pôle de Compétences Pharmacochimie, Université Mohammed V-Agdal, BP 1014 Avenue Ibn Batout, Rabat, Morocco; c Department of Chemistry, University of Malaya, 50603 Kuala Lumpur, Malaysia; d Chemistry Department, King Abdulaziz University, PO Box 80203 Jeddah, Saudi Arabia

## Abstract

In the title benzimidazolone, C_27_H_38_N_2_O_11_, which has *N*-bound glycosidic units, all five-membered O-heterocyclic rings adopt envelope conformations [for the outer rings, the C atom with the dimethyl groups represents the flap atom]. The two glycosidic units are related by a non-crystallographic twofold rotation axis that passes through the carbonyl portion. In the mol­ecular structure, the hy­droxy groups are hydrogen-bond donors to the carbonyl O atom. Weak inter­molecular C—H⋯O hydrogen bonding is present in the crystal structure.

## Related literature

For background to benzimidazolo­nes, see: Lakhrissi *et al.* (2011 ▶).
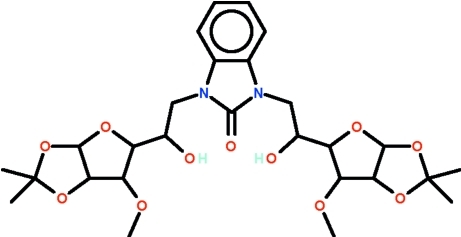


## Experimental

### 

#### Crystal data

C_27_H_38_N_2_O_11_
*M* *_r_* = 566.59
Monoclinic, 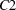

*a* = 30.5551 (8) Å
*b* = 5.3132 (1) Å
*c* = 17.8757 (5) Å
β = 98.267 (2)°
*V* = 2871.88 (12) Å^3^
*Z* = 4
Mo *K*α radiation
μ = 0.10 mm^−1^
*T* = 296 K
0.42 × 0.17 × 0.12 mm

#### Data collection

Bruker APEX DUO diffractometer
30976 measured reflections
3861 independent reflections
2734 reflections with *I* > 2σ(*I*)
*R*_int_ = 0.034

#### Refinement

*R*[*F* ^2^ > 2σ(*F* ^2^)] = 0.044
*wR*(*F* ^2^) = 0.134
*S* = 1.01
3861 reflections
363 parameters
1 restraint
H-atom parameters constrained
Δρ_max_ = 0.17 e Å^−3^
Δρ_min_ = −0.16 e Å^−3^

### 

Data collection: *APEX2* (Bruker, 2010 ▶); cell refinement: *SAINT* (Bruker, 2010 ▶); data reduction: *SAINT*; program(s) used to solve structure: *SHELXS97* (Sheldrick, 2008 ▶); program(s) used to refine structure: *SHELXL97* (Sheldrick, 2008 ▶); molecular graphics: *X-SEED* (Barbour, 2001 ▶); software used to prepare material for publication: *publCIF* (Westrip, 2010 ▶).

## Supplementary Material

Crystal structure: contains datablock(s) global, I. DOI: 10.1107/S160053681105625X/xu5430sup1.cif

Structure factors: contains datablock(s) I. DOI: 10.1107/S160053681105625X/xu5430Isup2.hkl

Supplementary material file. DOI: 10.1107/S160053681105625X/xu5430Isup3.cml

Additional supplementary materials:  crystallographic information; 3D view; checkCIF report

## Figures and Tables

**Table 1:** Hydrogen-bond geometry (Å, °)

*D*—H⋯*A*	*D*—H	H⋯*A*	*D*⋯*A*	*D*—H⋯*A*
O5—H5⋯O6	0.82	2.22	2.981 (3)	155
O7—H7⋯O6	0.82	2.30	2.946 (3)	136
C7—H7*A*⋯O4^i^	0.98	2.36	3.165 (4)	139
C21—H21⋯O10^ii^	0.98	2.32	3.103 (3)	136

Symmetry codes: (i) 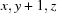; (ii) 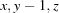.

**Fig. 1.**
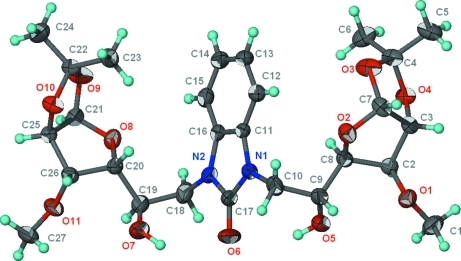
Thermal ellipsoid plot (Barbour, 2001) of C27H38N2O11 at the 30% probability level; hydrogen atoms are drawn as spheres of arbitrary radius.

## supplementary crystallographic information

### Comment

Benzimidazolones are heterocyclic compounds having pharmaceutical properties; benzimidazolones with glycosidic units showed surface-active properties (Lakhrissi *et al.*, 2011). Following our interest in benzimidazolone-based pharmaceutical compounds, we have synthesized the title compound (Scheme I) by the reaction of benzimidazol-2(3*H*)-one and 5,6-anhydro-1,2-*O*-isopropylidene-α-D-glucofuranose in the presence of potassium carbonate. The title compound (Scheme I) has all five-membered rings adopting envelope conformations (Fig. 1). The two units are related by a non-crystallographic two-fold rotation axis that passes through the carbonyl portion; the hydroxyl groups are hydrogen-bond donors to the carbonyl O atom (Table 1).

### Experimental

To benzimidazol-2(3*H*)-one (0.50 g, 3.73 mmol) and potassium carbonate (1.13 g, 8.23 mmol) in a mixture of toluene-DMSO (4:1, v/v) was added 5,6-anhydro-1,2-*O*-isopropylidene-α-D-glucofuranose (1.61 g, 7.46 mmol). Stirring was continued for 24 h at 383 K. The reaction was monitored by thin layer chromatography. The solid material was removed by filtration and the filtrate was concentrated under reduced pressure. The residue was extracted with toluene-water; the organic layer was removed, washed with saturated aqueous sodium chloride and then dried over sodium sulfate. The crude product obtained was purified by silica gel chromatography by using acetone/hexane (1/4). The eluent was allowed to evaporate to afford the title compound as colorless crystals.

### Refinement

Hydrogen atoms were placed in calculated positions (C–H 0.93–0.97 and O–H 0.82 Å) and were included in the refinement in the riding model approximation, with *U*(H) set to 1.2–1.5*U*(C,O).

The absolute configuration was those of the reactant; 3092 Friedel pairs were merged. The (2 0 0), (-2 0 1), (2 0 1) and (0 0 1) reflections were omitted owing to bad disagreement.

### Crystal data

**Table:**

C_27_H_38_N_2_O_11_	*F*(000) = 1208
*M**_r_* = 566.59	*D*_x_ = 1.310 Mg m^−^^3^
Monoclinic, *C*2	Mo *K*α radiation, λ = 0.71073 Å
Hall symbol: C 2y	Cell parameters from 8431 reflections
*a* = 30.5551 (8) Å	θ = 2.3–22.4°
*b* = 5.3132 (1) Å	µ = 0.10 mm^−^^1^
*c* = 17.8757 (5) Å	*T* = 296 K
β = 98.267 (2)°	Prism, colorless
*V* = 2871.88 (12) Å^3^	0.42 × 0.17 × 0.12 mm
*Z* = 4	

### Data collection

**Table:**

Bruker APEX DUO diffractometer	2734 reflections with *I* > 2σ(*I*)
Radiation source: fine-focus sealed tube	*R*_int_ = 0.034
graphite	θ_max_ = 28.1°, θ_min_ = 2.3°
ω scans	*h* = −40→40
30976 measured reflections	*k* = −7→7
3861 independent reflections	*l* = −23→23

### Refinement

**Table:**

Refinement on *F*^2^	Primary atom site location: structure-invariant direct methods
Least-squares matrix: full	Secondary atom site location: difference Fourier map
*R*[*F*^2^ > 2σ(*F*^2^)] = 0.044	Hydrogen site location: inferred from neighbouring sites
*wR*(*F*^2^) = 0.134	H-atom parameters constrained
*S* = 1.01	*w* = 1/[σ^2^(*F*_o_^2^) + (0.0702*P*)^2^ + 0.7057*P*] where *P* = (*F*_o_^2^ + 2*F*_c_^2^)/3
3861 reflections	(Δ/σ)_max_ = 0.001
363 parameters	Δρ_max_ = 0.17 e Å^−^^3^
1 restraint	Δρ_min_ = −0.16 e Å^−^^3^

### Fractional atomic coordinates and isotropic or equivalent isotropic displacement parameters (Å^2^)

**Table:**

	*x*	*y*	*z*	*U*_iso_*/*U*_eq_	
O1	0.45026 (6)	0.5033 (5)	0.33539 (12)	0.0766 (6)	
O2	0.37502 (7)	0.7202 (4)	0.23328 (12)	0.0767 (6)	
O3	0.37553 (11)	0.6090 (5)	0.10928 (13)	0.0998 (9)	
O4	0.41260 (7)	0.2587 (4)	0.14954 (10)	0.0654 (5)	
O5	0.36385 (8)	0.3556 (9)	0.39848 (13)	0.1219 (14)	
H5	0.3436	0.3546	0.4241	0.183*	
O6	0.27339 (8)	0.3692 (13)	0.44238 (12)	0.174 (3)	
O7	0.17794 (8)	0.3550 (10)	0.45163 (15)	0.1362 (17)	
H7	0.2032	0.3342	0.4733	0.204*	
O8	0.11563 (7)	0.0500 (5)	0.29169 (12)	0.0794 (6)	
O9	0.06657 (10)	0.1535 (4)	0.18397 (12)	0.0876 (8)	
O10	0.05679 (8)	0.5151 (4)	0.24633 (11)	0.0710 (6)	
O11	0.07896 (7)	0.2187 (6)	0.42890 (11)	0.0880 (8)	
N1	0.27933 (7)	0.5088 (8)	0.32190 (13)	0.0886 (10)	
N2	0.23072 (8)	0.2129 (9)	0.33524 (13)	0.0946 (12)	
C1	0.47984 (12)	0.3388 (10)	0.3764 (2)	0.1010 (13)	
H1A	0.5000	0.4321	0.4123	0.152*	
H1B	0.4961	0.2501	0.3426	0.152*	
H1C	0.4639	0.2204	0.4028	0.152*	
C2	0.41922 (8)	0.3811 (6)	0.28051 (14)	0.0604 (6)	
H2	0.4188	0.1989	0.2889	0.072*	
C3	0.42988 (9)	0.4441 (5)	0.20226 (15)	0.0579 (6)	
H3	0.4615	0.4741	0.2019	0.070*	
C4	0.39090 (12)	0.3801 (6)	0.08371 (16)	0.0733 (8)	
C5	0.42325 (15)	0.4237 (11)	0.02859 (19)	0.1074 (13)	
H5A	0.4474	0.5248	0.0522	0.161*	
H5B	0.4086	0.5092	−0.0153	0.161*	
H5C	0.4343	0.2649	0.0139	0.161*	
C6	0.35169 (13)	0.2237 (9)	0.0517 (2)	0.1029 (13)	
H6A	0.3324	0.2036	0.0892	0.154*	
H6B	0.3616	0.0615	0.0375	0.154*	
H6C	0.3361	0.3062	0.0081	0.154*	
C7	0.40167 (11)	0.6756 (6)	0.17787 (17)	0.0686 (8)	
H7A	0.4203	0.8220	0.1712	0.082*	
C8	0.37374 (9)	0.4957 (7)	0.27796 (15)	0.0659 (7)	
H8	0.3515	0.3799	0.2523	0.079*	
C9	0.36052 (10)	0.5741 (10)	0.35228 (17)	0.0879 (12)	
H9	0.3818	0.6990	0.3756	0.105*	
C10	0.31454 (10)	0.6894 (11)	0.3438 (2)	0.1013 (15)	
H10A	0.3102	0.7661	0.3914	0.122*	
H10B	0.3127	0.8215	0.3060	0.122*	
C11	0.25900 (8)	0.4453 (8)	0.24966 (15)	0.0717 (9)	
C12	0.26519 (10)	0.5351 (10)	0.17970 (17)	0.0886 (12)	
H12	0.2857	0.6606	0.1742	0.106*	
C13	0.23948 (11)	0.4296 (13)	0.11848 (18)	0.1071 (17)	
H13	0.2425	0.4866	0.0703	0.128*	
C14	0.20961 (12)	0.2436 (13)	0.12611 (19)	0.1110 (18)	
H14	0.1932	0.1759	0.0830	0.133*	
C15	0.20314 (10)	0.1534 (10)	0.19590 (19)	0.0950 (13)	
H15	0.1826	0.0275	0.2010	0.114*	
C16	0.22854 (9)	0.2586 (9)	0.25767 (15)	0.0759 (10)	
C17	0.26215 (10)	0.3639 (13)	0.37379 (17)	0.1112 (19)	
C18	0.20542 (11)	0.0311 (13)	0.3720 (2)	0.1167 (18)	
H18A	0.2247	−0.0478	0.4132	0.140*	
H18B	0.1947	−0.0992	0.3360	0.140*	
C19	0.16587 (10)	0.1509 (11)	0.40316 (19)	0.1004 (15)	
H19	0.1520	0.0222	0.4312	0.120*	
C20	0.13151 (8)	0.2522 (7)	0.34121 (15)	0.0691 (8)	
H20	0.1451	0.3805	0.3125	0.083*	
C21	0.07154 (9)	0.0921 (5)	0.26029 (16)	0.0603 (7)	
H21	0.0534	−0.0549	0.2683	0.072*	
C22	0.06186 (10)	0.4169 (5)	0.17386 (16)	0.0605 (7)	
C23	0.10223 (10)	0.5292 (8)	0.14949 (18)	0.0817 (9)	
H23A	0.1275	0.4909	0.1862	0.123*	
H23B	0.0987	0.7084	0.1452	0.123*	
H23C	0.1065	0.4606	0.1014	0.123*	
C24	0.02076 (11)	0.4703 (10)	0.1193 (2)	0.0961 (12)	
H24A	−0.0043	0.3961	0.1377	0.144*	
H24B	0.0238	0.4000	0.0708	0.144*	
H24C	0.0166	0.6489	0.1145	0.144*	
C25	0.05565 (8)	0.3204 (5)	0.29936 (14)	0.0545 (6)	
H25	0.0260	0.2961	0.3132	0.065*	
C26	0.09055 (9)	0.3618 (6)	0.36827 (14)	0.0633 (7)	
H26	0.0942	0.5405	0.3812	0.076*	
C27	0.06435 (14)	0.3616 (13)	0.48528 (18)	0.1196 (19)	
H27A	0.0569	0.2525	0.5244	0.179*	
H27B	0.0387	0.4561	0.4644	0.179*	
H27C	0.0873	0.4752	0.5062	0.179*	

### Atomic displacement parameters (Å^2^)

**Table:**

	*U*^11^	*U*^22^	*U*^33^	*U*^12^	*U*^13^	*U*^23^
O1	0.0660 (11)	0.0826 (16)	0.0778 (12)	−0.0040 (12)	−0.0015 (9)	−0.0167 (13)
O2	0.0851 (13)	0.0624 (13)	0.0865 (13)	0.0135 (11)	0.0260 (11)	−0.0062 (11)
O3	0.161 (2)	0.0603 (14)	0.0740 (14)	0.0439 (16)	0.0026 (15)	0.0019 (12)
O4	0.0904 (13)	0.0398 (10)	0.0644 (11)	0.0097 (10)	0.0062 (9)	−0.0033 (9)
O5	0.0779 (14)	0.226 (4)	0.0636 (13)	0.002 (2)	0.0151 (11)	0.017 (2)
O6	0.0872 (15)	0.380 (8)	0.0505 (11)	−0.064 (3)	−0.0020 (10)	0.015 (3)
O7	0.0725 (14)	0.258 (5)	0.0744 (14)	−0.031 (2)	−0.0014 (11)	−0.008 (3)
O8	0.0669 (12)	0.0758 (15)	0.0943 (15)	0.0146 (11)	0.0079 (10)	−0.0080 (13)
O9	0.151 (2)	0.0438 (12)	0.0662 (13)	0.0104 (14)	0.0109 (13)	−0.0066 (10)
O10	0.1105 (16)	0.0384 (10)	0.0670 (11)	0.0061 (11)	0.0227 (11)	0.0008 (9)
O11	0.0892 (13)	0.113 (2)	0.0667 (12)	−0.0176 (15)	0.0283 (11)	0.0105 (14)
N1	0.0554 (12)	0.155 (3)	0.0552 (13)	−0.0049 (18)	0.0080 (10)	−0.0127 (19)
N2	0.0527 (12)	0.166 (4)	0.0640 (14)	0.0023 (19)	0.0037 (11)	0.018 (2)
C1	0.079 (2)	0.126 (4)	0.091 (2)	−0.011 (2)	−0.0100 (17)	0.020 (3)
C2	0.0650 (14)	0.0552 (16)	0.0605 (14)	−0.0068 (13)	0.0073 (11)	−0.0038 (13)
C3	0.0695 (15)	0.0435 (14)	0.0632 (15)	−0.0036 (12)	0.0179 (12)	−0.0056 (12)
C4	0.109 (2)	0.0485 (16)	0.0623 (16)	0.0191 (17)	0.0128 (16)	0.0015 (14)
C5	0.144 (3)	0.113 (3)	0.0684 (19)	−0.011 (3)	0.028 (2)	0.000 (2)
C6	0.108 (3)	0.093 (3)	0.099 (2)	0.008 (3)	−0.016 (2)	−0.001 (2)
C7	0.095 (2)	0.0382 (14)	0.0783 (18)	0.0023 (14)	0.0306 (16)	−0.0018 (13)
C8	0.0619 (14)	0.078 (2)	0.0587 (15)	−0.0090 (15)	0.0121 (11)	−0.0092 (15)
C9	0.0644 (16)	0.137 (4)	0.0619 (17)	−0.011 (2)	0.0087 (14)	−0.027 (2)
C10	0.0701 (18)	0.158 (4)	0.079 (2)	−0.004 (2)	0.0204 (16)	−0.047 (3)
C11	0.0517 (13)	0.111 (3)	0.0517 (14)	0.0174 (17)	0.0039 (11)	−0.0067 (16)
C12	0.0699 (17)	0.131 (4)	0.0655 (17)	0.020 (2)	0.0112 (14)	0.006 (2)
C13	0.0747 (19)	0.190 (5)	0.0553 (16)	0.020 (3)	0.0050 (15)	0.004 (3)
C14	0.0730 (19)	0.190 (5)	0.0662 (19)	0.016 (3)	−0.0017 (16)	−0.041 (3)
C15	0.0607 (16)	0.138 (4)	0.085 (2)	0.015 (2)	0.0037 (15)	−0.026 (3)
C16	0.0495 (13)	0.116 (3)	0.0612 (15)	0.0206 (17)	0.0051 (11)	−0.0022 (18)
C17	0.0542 (15)	0.222 (6)	0.0568 (16)	−0.004 (3)	0.0049 (13)	0.004 (3)
C18	0.0664 (18)	0.176 (5)	0.108 (3)	0.025 (3)	0.0119 (18)	0.061 (3)
C19	0.0643 (17)	0.161 (5)	0.076 (2)	−0.003 (2)	0.0099 (16)	0.036 (3)
C20	0.0533 (13)	0.094 (2)	0.0590 (14)	−0.0128 (16)	0.0066 (11)	0.0080 (16)
C21	0.0638 (15)	0.0421 (14)	0.0735 (17)	−0.0030 (12)	0.0042 (13)	0.0028 (13)
C22	0.0768 (16)	0.0463 (15)	0.0579 (14)	0.0066 (13)	0.0084 (12)	−0.0020 (12)
C23	0.0849 (19)	0.085 (2)	0.0790 (19)	0.0100 (19)	0.0228 (16)	0.0095 (19)
C24	0.084 (2)	0.107 (3)	0.092 (2)	0.002 (2)	−0.0052 (17)	0.009 (2)
C25	0.0579 (13)	0.0434 (14)	0.0628 (14)	−0.0035 (11)	0.0108 (11)	0.0028 (11)
C26	0.0664 (14)	0.0660 (18)	0.0590 (14)	−0.0146 (14)	0.0148 (11)	−0.0018 (14)
C27	0.117 (3)	0.187 (6)	0.0603 (18)	0.040 (4)	0.0316 (19)	0.019 (3)

### Geometric parameters (Å, °)

**Table:**

O1—C1	1.389 (5)	C6—H6C	0.9600
O1—C2	1.420 (3)	C7—H7A	0.9800
O2—C7	1.390 (4)	C8—C9	1.502 (4)
O2—C8	1.439 (4)	C8—H8	0.9800
O3—C4	1.404 (4)	C9—C10	1.520 (5)
O3—C7	1.409 (4)	C9—H9	0.9800
O4—C3	1.412 (3)	C10—H10A	0.9700
O4—C4	1.420 (3)	C10—H10B	0.9700
O5—C9	1.420 (6)	C11—C12	1.377 (4)
O5—H5	0.8200	C11—C16	1.381 (5)
O6—C17	1.225 (3)	C12—C13	1.372 (5)
O7—C19	1.404 (7)	C12—H12	0.9300
O7—H7	0.8200	C13—C14	1.365 (8)
O8—C21	1.401 (3)	C13—H13	0.9300
O8—C20	1.432 (4)	C14—C15	1.377 (6)
O9—C21	1.389 (4)	C14—H14	0.9300
O9—C22	1.416 (4)	C15—C16	1.374 (5)
O10—C25	1.407 (3)	C15—H15	0.9300
O10—C22	1.426 (3)	C18—C19	1.539 (6)
O11—C27	1.386 (5)	C18—H18A	0.9700
O11—C26	1.410 (3)	C18—H18B	0.9700
N1—C17	1.367 (6)	C19—C20	1.511 (4)
N1—C11	1.392 (3)	C19—H19	0.9800
N1—C10	1.452 (5)	C20—C26	1.521 (4)
N2—C17	1.360 (6)	C20—H20	0.9800
N2—C16	1.400 (4)	C21—C25	1.514 (4)
N2—C18	1.452 (6)	C21—H21	0.9800
C1—H1A	0.9600	C22—C23	1.491 (5)
C1—H1B	0.9600	C22—C24	1.502 (4)
C1—H1C	0.9600	C23—H23A	0.9600
C2—C8	1.512 (4)	C23—H23B	0.9600
C2—C3	1.518 (4)	C23—H23C	0.9600
C2—H2	0.9800	C24—H24A	0.9600
C3—C7	1.529 (4)	C24—H24B	0.9600
C3—H3	0.9800	C24—H24C	0.9600
C4—C6	1.502 (5)	C25—C26	1.525 (4)
C4—C5	1.511 (5)	C25—H25	0.9800
C5—H5A	0.9600	C26—H26	0.9800
C5—H5B	0.9600	C27—H27A	0.9600
C5—H5C	0.9600	C27—H27B	0.9600
C6—H6A	0.9600	C27—H27C	0.9600
C6—H6B	0.9600		
			
C1—O1—C2	113.3 (3)	C13—C12—C11	116.5 (4)
C7—O2—C8	108.6 (2)	C13—C12—H12	121.8
C4—O3—C7	109.1 (3)	C11—C12—H12	121.8
C3—O4—C4	108.8 (2)	C12—C13—C14	122.0 (4)
C9—O5—H5	109.5	C12—C13—H13	119.0
C19—O7—H7	109.5	C14—C13—H13	119.0
C21—O8—C20	110.4 (2)	C15—C14—C13	121.8 (4)
C21—O9—C22	110.6 (2)	C15—C14—H14	119.1
C25—O10—C22	111.0 (2)	C13—C14—H14	119.1
C27—O11—C26	114.0 (4)	C14—C15—C16	116.6 (4)
C17—N1—C11	109.2 (3)	C14—C15—H15	121.7
C17—N1—C10	122.1 (3)	C16—C15—H15	121.7
C11—N1—C10	128.6 (3)	C15—C16—C11	121.3 (3)
C17—N2—C16	109.2 (3)	C15—C16—N2	131.8 (4)
C17—N2—C18	123.1 (3)	C11—C16—N2	106.9 (3)
C16—N2—C18	127.7 (4)	O6—C17—N2	126.6 (5)
O1—C1—H1A	109.5	O6—C17—N1	125.9 (5)
O1—C1—H1B	109.5	N2—C17—N1	107.5 (3)
H1A—C1—H1B	109.5	N2—C18—C19	112.7 (5)
O1—C1—H1C	109.5	N2—C18—H18A	109.1
H1A—C1—H1C	109.5	C19—C18—H18A	109.1
H1B—C1—H1C	109.5	N2—C18—H18B	109.1
O1—C2—C8	110.9 (2)	C19—C18—H18B	109.1
O1—C2—C3	109.0 (2)	H18A—C18—H18B	107.8
C8—C2—C3	101.6 (2)	O7—C19—C20	105.7 (4)
O1—C2—H2	111.6	O7—C19—C18	113.1 (3)
C8—C2—H2	111.6	C20—C19—C18	112.4 (3)
C3—C2—H2	111.6	O7—C19—H19	108.5
O4—C3—C2	110.6 (2)	C20—C19—H19	108.5
O4—C3—C7	103.3 (2)	C18—C19—H19	108.5
C2—C3—C7	104.7 (2)	O8—C20—C19	108.9 (3)
O4—C3—H3	112.6	O8—C20—C26	105.2 (2)
C2—C3—H3	112.6	C19—C20—C26	114.8 (2)
C7—C3—H3	112.6	O8—C20—H20	109.2
O3—C4—O4	105.2 (2)	C19—C20—H20	109.2
O3—C4—C6	108.5 (3)	C26—C20—H20	109.2
O4—C4—C6	108.4 (3)	O9—C21—O8	113.2 (3)
O3—C4—C5	111.1 (3)	O9—C21—C25	105.5 (2)
O4—C4—C5	109.8 (3)	O8—C21—C25	107.3 (2)
C6—C4—C5	113.4 (3)	O9—C21—H21	110.2
C4—C5—H5A	109.5	O8—C21—H21	110.2
C4—C5—H5B	109.5	C25—C21—H21	110.2
H5A—C5—H5B	109.5	O9—C22—O10	105.6 (2)
C4—C5—H5C	109.5	O9—C22—C23	111.1 (3)
H5A—C5—H5C	109.5	O10—C22—C23	108.8 (2)
H5B—C5—H5C	109.5	O9—C22—C24	109.3 (3)
C4—C6—H6A	109.5	O10—C22—C24	109.3 (3)
C4—C6—H6B	109.5	C23—C22—C24	112.6 (3)
H6A—C6—H6B	109.5	C22—C23—H23A	109.5
C4—C6—H6C	109.5	C22—C23—H23B	109.5
H6A—C6—H6C	109.5	H23A—C23—H23B	109.5
H6B—C6—H6C	109.5	C22—C23—H23C	109.5
O2—C7—O3	110.2 (3)	H23A—C23—H23C	109.5
O2—C7—C3	107.3 (2)	H23B—C23—H23C	109.5
O3—C7—C3	105.8 (2)	C22—C24—H24A	109.5
O2—C7—H7A	111.1	C22—C24—H24B	109.5
O3—C7—H7A	111.1	H24A—C24—H24B	109.5
C3—C7—H7A	111.1	C22—C24—H24C	109.5
O2—C8—C9	107.0 (3)	H24A—C24—H24C	109.5
O2—C8—C2	104.6 (2)	H24B—C24—H24C	109.5
C9—C8—C2	116.6 (2)	O10—C25—C21	103.75 (19)
O2—C8—H8	109.4	O10—C25—C26	110.9 (2)
C9—C8—H8	109.4	C21—C25—C26	104.8 (2)
C2—C8—H8	109.4	O10—C25—H25	112.3
O5—C9—C8	106.4 (4)	C21—C25—H25	112.3
O5—C9—C10	112.1 (3)	C26—C25—H25	112.3
C8—C9—C10	112.7 (3)	O11—C26—C20	110.3 (3)
O5—C9—H9	108.5	O11—C26—C25	108.6 (2)
C8—C9—H9	108.5	C20—C26—C25	101.5 (2)
C10—C9—H9	108.5	O11—C26—H26	112.0
N1—C10—C9	113.5 (4)	C20—C26—H26	112.0
N1—C10—H10A	108.9	C25—C26—H26	112.0
C9—C10—H10A	108.9	O11—C27—H27A	109.5
N1—C10—H10B	108.9	O11—C27—H27B	109.5
C9—C10—H10B	108.9	H27A—C27—H27B	109.5
H10A—C10—H10B	107.7	O11—C27—H27C	109.5
C12—C11—C16	121.7 (3)	H27A—C27—H27C	109.5
C12—C11—N1	131.1 (4)	H27B—C27—H27C	109.5
C16—C11—N1	107.2 (3)		
			
C1—O1—C2—C8	139.7 (3)	N1—C11—C16—N2	0.4 (4)
C1—O1—C2—C3	−109.3 (3)	C17—N2—C16—C15	178.7 (4)
C4—O4—C3—C2	133.8 (2)	C18—N2—C16—C15	0.5 (7)
C4—O4—C3—C7	22.3 (3)	C17—N2—C16—C11	−1.0 (4)
O1—C2—C3—O4	156.9 (2)	C18—N2—C16—C11	−179.3 (4)
C8—C2—C3—O4	−86.0 (3)	C16—N2—C17—O6	−178.3 (6)
O1—C2—C3—C7	−92.5 (3)	C18—N2—C17—O6	0.0 (9)
C8—C2—C3—C7	24.6 (3)	C16—N2—C17—N1	1.3 (5)
C7—O3—C4—O4	24.3 (4)	C18—N2—C17—N1	179.6 (4)
C7—O3—C4—C6	140.1 (3)	C11—N1—C17—O6	178.6 (6)
C7—O3—C4—C5	−94.5 (3)	C10—N1—C17—O6	0.8 (8)
C3—O4—C4—O3	−29.5 (3)	C11—N1—C17—N2	−1.0 (5)
C3—O4—C4—C6	−145.4 (3)	C10—N1—C17—N2	−178.8 (4)
C3—O4—C4—C5	90.1 (3)	C17—N2—C18—C19	82.3 (5)
C8—O2—C7—O3	96.9 (3)	C16—N2—C18—C19	−99.7 (5)
C8—O2—C7—C3	−17.9 (3)	N2—C18—C19—O7	−54.2 (4)
C4—O3—C7—O2	−126.3 (3)	N2—C18—C19—C20	65.3 (5)
C4—O3—C7—C3	−10.5 (3)	C21—O8—C20—C19	149.5 (2)
O4—C3—C7—O2	110.5 (3)	C21—O8—C20—C26	25.9 (3)
C2—C3—C7—O2	−5.3 (3)	O7—C19—C20—O8	−176.7 (2)
O4—C3—C7—O3	−7.2 (3)	C18—C19—C20—O8	59.5 (5)
C2—C3—C7—O3	−123.0 (3)	O7—C19—C20—C26	−59.0 (4)
C7—O2—C8—C9	158.6 (2)	C18—C19—C20—C26	177.2 (4)
C7—O2—C8—C2	34.3 (3)	C22—O9—C21—O8	−99.7 (3)
O1—C2—C8—O2	80.2 (3)	C22—O9—C21—C25	17.3 (4)
C3—C2—C8—O2	−35.5 (3)	C20—O8—C21—O9	108.5 (3)
O1—C2—C8—C9	−37.8 (4)	C20—O8—C21—C25	−7.5 (3)
C3—C2—C8—C9	−153.5 (3)	C21—O9—C22—O10	−9.3 (4)
O2—C8—C9—O5	−174.1 (2)	C21—O9—C22—C23	108.4 (3)
C2—C8—C9—O5	−57.4 (4)	C21—O9—C22—C24	−126.8 (3)
O2—C8—C9—C10	62.8 (4)	C25—O10—C22—O9	−3.4 (3)
C2—C8—C9—C10	179.4 (4)	C25—O10—C22—C23	−122.7 (3)
C17—N1—C10—C9	85.0 (4)	C25—O10—C22—C24	114.0 (3)
C11—N1—C10—C9	−92.3 (5)	C22—O10—C25—C21	13.4 (3)
O5—C9—C10—N1	−50.1 (4)	C22—O10—C25—C26	125.3 (2)
C8—C9—C10—N1	69.8 (5)	O9—C21—C25—O10	−18.4 (3)
C17—N1—C11—C12	−179.2 (4)	O8—C21—C25—O10	102.5 (3)
C10—N1—C11—C12	−1.6 (7)	O9—C21—C25—C26	−134.8 (2)
C17—N1—C11—C16	0.4 (4)	O8—C21—C25—C26	−13.8 (3)
C10—N1—C11—C16	178.0 (4)	C27—O11—C26—C20	141.0 (3)
C16—C11—C12—C13	0.1 (6)	C27—O11—C26—C25	−108.6 (3)
N1—C11—C12—C13	179.7 (4)	O8—C20—C26—O11	82.3 (3)
C11—C12—C13—C14	−0.7 (7)	C19—C20—C26—O11	−37.4 (4)
C12—C13—C14—C15	0.9 (8)	O8—C20—C26—C25	−32.7 (3)
C13—C14—C15—C16	−0.5 (7)	C19—C20—C26—C25	−152.4 (3)
C14—C15—C16—C11	−0.1 (6)	O10—C25—C26—O11	160.3 (2)
C14—C15—C16—N2	−179.8 (4)	C21—C25—C26—O11	−88.4 (3)
C12—C11—C16—C15	0.3 (5)	O10—C25—C26—C20	−83.4 (3)
N1—C11—C16—C15	−179.4 (3)	C21—C25—C26—C20	27.9 (3)
C12—C11—C16—N2	−180.0 (3)		

### Hydrogen-bond geometry (Å, °)

**Table:**

*D*—H···*A*	*D*—H	H···*A*	*D*···*A*	*D*—H···*A*
O5—H5···O6	0.82	2.22	2.981 (3)	155
O7—H7···O6	0.82	2.30	2.946 (3)	136
C7—H7A···O4^i^	0.98	2.36	3.165 (4)	139
C21—H21···O10^ii^	0.98	2.32	3.103 (3)	136

Symmetry codes: (i) *x*, *y*+1, *z*; (ii) *x*, *y*−1, *z*.
